# Isolation of extracellular vesicles improves the detection of mutant DNA from plasma of metastatic melanoma patients

**DOI:** 10.1038/s41598-020-72834-6

**Published:** 2020-09-25

**Authors:** Davide Zocco, Simona Bernardi, Mauro Novelli, Chiara Astrua, Paolo Fava, Natasa Zarovni, Francesco M. Carpi, Laura Bianciardi, Ottavia Malavenda, Pietro Quaglino, Chiara Foroni, Domenico Russo, Antonio Chiesi, Maria Teresa Fierro

**Affiliations:** 1 Exosomics SpA, Siena, Italy; 2 Laboratorio Immunopatologia Cutanea, Clinica Dermatologica, Dipartimento Scienze Mediche, Università di Torino, Turin, Italy; 3 Azienda Ospedaliera Città della Salute e della Scienza di Torino, Turin, Italy; 4 Lab. CREA - A.I.L., Spedali Civili di Brescia, Brescia, Italy; 5 Chair of Hematology - Unit of Bone Marrow Transplantation, University of Brescia, Brescia, Italy

**Keywords:** Melanoma, Tumour biomarkers

## Abstract

Detection of *BRAF*^V600E^ within cell free tumor DNA (ctDNA) is emerging as a promising means to improve patients’ stratification or enable BRAF inhibitor (BRAFi) therapeutic monitoring in a minimally invasive manner. Here, we investigated whether extracellular vesicle-(EV)-associated-DNA (EV-DNA) has value as an alternative source of circulating *BRAF*^V600E^. To do so, we identified a clinical practice-compatible protocol for the isolation of EV-DNA and assessed *BRAF* gene status on plasma samples from metastatic melanoma patients at the beginning and during BRAFi therapy. This protocol uses a peptide with high affinity for EVs and it has been found to recover more mutant DNA from plasma than standard ultracentrifugation. Molecular analyses revealed that mutant DNA is largely unprotected from nuclease digestion, interacting with the outer side of the EV membrane or directly with the peptide. When used on clinical samples, we found that the protocol improves the detection of *BRAF*^V600E^ gene copies in comparison to the reference protocol for ctDNA isolation. Taken together, these findings indicate that EVs are a promising source of mutant DNA and should be considered for the development of next-generation liquid biopsy approaches.

## Introduction

Melanoma is the most serious and aggressive form of skin cancer, accounting for 79% of all skin cancer deaths^1^. Nearly 60% of melanoma patients’ tumors harbor mutations in the *BRAF* gene that correlate with aggressive tumor phenotypes and poor prognosis^1^. In up to 90% of *BRAF* mutation cases, thymine is substituted by adenine at nucleotide 1799, leading to valine (V) being substituted with glutamate (E) at codon 600 (*BRAF*^V600E^; 1). Selective inhibition of B-Raf with vemurafenib (Zelboraf; Roche) or dabrafenib (Tafinlar; Novartis) currently used in association with comibenitib (Cotellic; Roche) and trametinib (Mekinist; Novartis), has revolutionized the treatment of unresectable or metastatic melanoma (MM) patients bearing *BRAF* mutations^2,3^. These patients are routinely identified with molecular diagnostic testing (eg. sequencing, PCR) using DNA specimens obtained from primary and/or metastatic tumor tissues. However, this procedure has two major shortcomings: (a) it is invasive and thus cannot be used to re-assess or monitor the *BRAF* gene status overtime; (b) a significant number of mutation-positive patients are missed due to tumor genomic heterogeneity^4^.

Recently, liquid-biopsy approaches based on circulating cell free DNA (cfDNA) have been proposed to circumvent these challenges^5^. Circulating tumor-derived DNA (ctDNA) can be isolated from blood in a minimally invasive manner and may carry actionable mutations coming from distant or inaccessible tumor sites^5,6^. Detection of *BRAF*^V600E^ mutation in ctDNA has been reported as a clinically useful means to identify MM patients with unfavorable prognostic outlook or to monitor BRAF inhibitor (BRAFi) treatment response^7,8^. Since *BRAF*^V600E^-bearing ctDNA represents only a minor fraction of total circulating DNA and cannot be selectively enriched, sensitive analytical techniques, such as droplet digital PCR (ddPCR), have been used to detect mutation allelic frequencies down to 0.1% or below^8^. To further improve diagnostic sensitivity, selective enrichment of tumor derived DNA within different plasma fractions has been proposed, especially when sample volume is limited^9^.

Extracellular vesicles (EVs) are lipid membrane vesicles released by most cells in physiological and/or pathological processes^10,11^. EVs are commonly classified based on their size and biogenesis. Large EVs, such as oncosomes and apoptotic bodies, have a diameter ranging from 1 to 4 μM^10,11^. Microvesicles and exosomes are smaller EVs with diameter ranging from 100 to 1000 nm and 30 to 120 nm, respectively. All but apoptotic bodies are released in circulation by living cells but their biogenesis may differ. Oncosomes and microvesicles originate from the budding of the plasma membrane while exosomes are generated in the endosomal compartment within the cell and are released from the multi vesicular bodies (MVB)^10,11^.

EVs have been described as key mediators of cancer cell communication and metastatic niche formation^12,13^. Cancer-derived EVs have been shown to carry tumor protein antigens^14–16^, specific RNA signatures^17,18^, single strand DNA^19^ and, more recently, double strand DNA with actionable mutations^20,21^. High prevalence of *KRAS* mutation was found in EV-associated DNA (EV-DNA) from early stage pancreatic cancer patients, further supporting the role of EVs as a carrier of clinically valuable genomic variants^22^.

In most of the aforementioned reports, EVs were isolated using the standard ultracentrifugation-based protocol originally published by Théry et al.^23^ and no further efforts were made to develop protocols free from the use of capital equipment and lengthy centrifugation steps. Only recently, new isolation methods based on filtration, chemical carriers or affinity ligands have become commercially available, but they have not been extensively evaluated on clinical samples^23,24^.

Therefore, the aim of this study was twofold: (a) to identify an ultracentrifuge-free, user-friendly protocol for the isolation of plasma EV-DNA in the clinical setting and (b) to assess the benefit of detecting *BRAF*^V600E^ in EV-DNA in addition to ctDNA for the stratification and BRAFi therapy monitoring of MM patients.

## Results

### Selection of a protocol for isolation of EV-associated DNA

**Figure 1:**
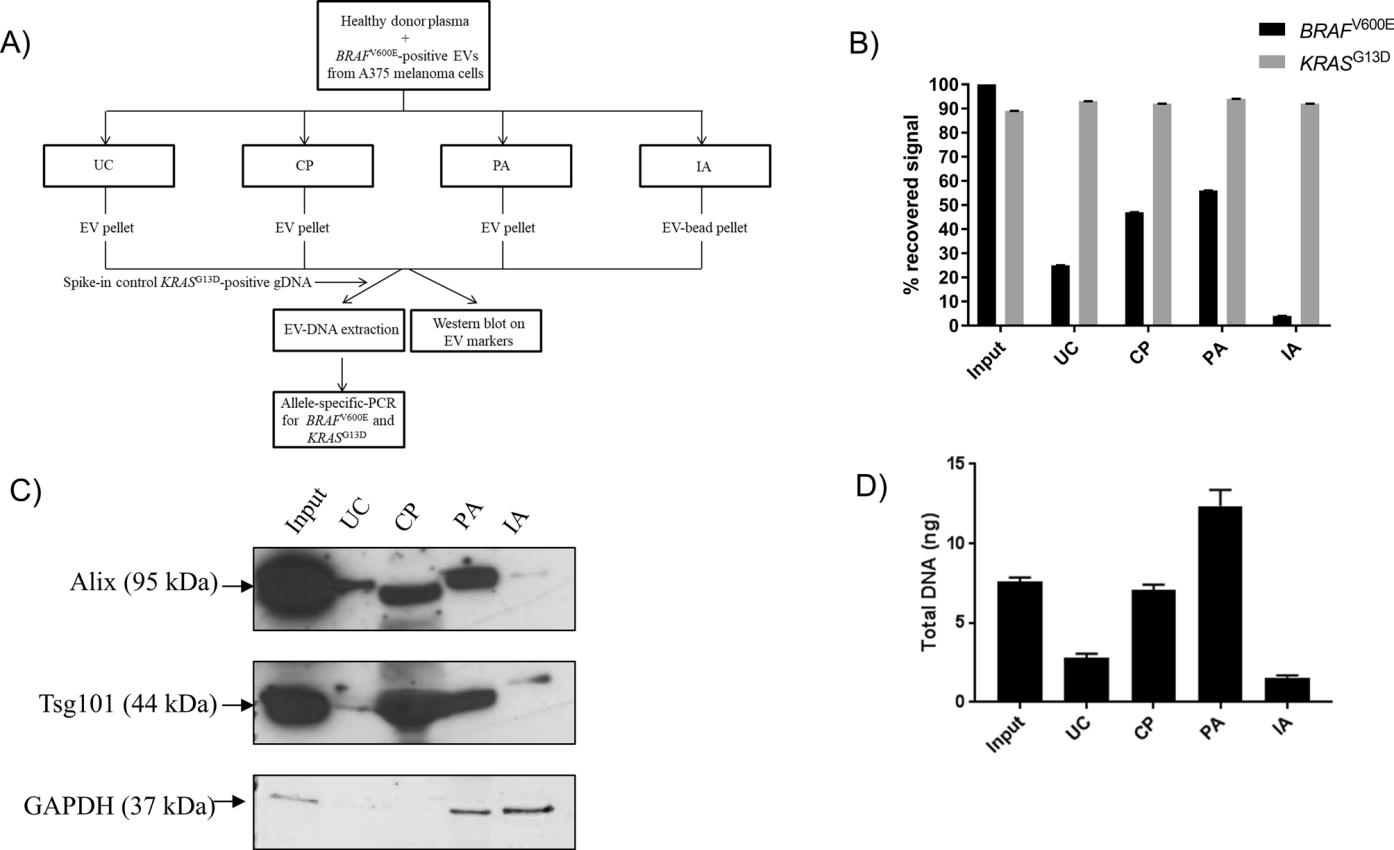
Selection of a protocol for isolation of EV-associated DNA (EV-DNA). (**A**) Experiment workflow; (**B**) Recovery of *BRAF*^V600E^-positive extracellular vesicles (EVs) from healthy donor plasma samples with ultracentrifugation (UC), chemical precipitation (CP), peptide affinity (PA) isolation and immunoaffinity (IA) isolation. Following DNA extraction, both mutant and wild type *BRAF* and *KRAS* genes were detected by allele-specific quantitative PCR (AS-QPCR). Results are representative of four independent experiments. (**C**) Western blot analysis of exosome markers Alix, Tsg101 and GAPDH after UC, CP, PA and IA isolation; blot is representative of three independent experiments. The original full-length blot is available as supplementary information (Supplementary Figure 6). (**D**) Quantitation of DNA extracted from UC- CP-, PA- and IA-derived pellets by fluorimetric assay.

Several methods have been developed to isolate EVs from biological fluids without the use of ultracentrifugation (UC) (^24,25^; Suppl. Table 1). Chemical precipitation (CP), peptide-based affinity isolation (PA) and antibody-based affinity capture (IA) were selected for their fast turnaround time, ease-of-use and ability to capture EVs from as low as 0.5 ml of plasma (Suppl. Table 1). These methods were compared to UC for their ability to capture *BRAF*^V600E^–positive EVs derived from a melanoma cell line and spiked into healthy donor (HD) plasma (Fig. 1A). Genomic DNA containing *KRAS*^G13D^ was added to the lysis buffer to control for DNA extraction and PCR amplification efficiency (Fig. 1A). Both *BRAF*^V600E^ and *KRAS*^G13D^ were detected by allele-specific quantitative PCR (qPCR) with a LOD of 0.1% (Suppl. Fig. 1A–B). We found that PA recovered more EV-derived *BRAF*^V600E^ mutation than CP, UC and IA using anti-tetraspanin-CD63 and anti-CD9-antibody-coated beads (56%, 47%, 25% or 4% respectively; Fig. 1B). Neither PA nor other isolation methods significantly affected DNA extraction and PCR amplification efficiency as the recovery of *KRAS*^G13D^ signal was found to be similar among samples (92% ± 3%; Fig. 1B). Consistent with these data, PA and CP protocols yielded the highest amount of canonical EV proteins Alix and Tsg101, confirming the efficient capture of EVs from plasma (Fig. 1C). Surprisingly, GAPDH protein was found in the PA but not in the CP pellet, suggesting that these methods isolate different sample fractions (Fig. 1C). While both protocols isolated only a fraction of the EV proteins in comparison to the input EV sample (Fig. 1C), PA recovered more DNA than any of the examined samples, including the input due to the isolation of plasma-derived cfDNA (PA = 12.36 ng/ml, input = 7.62 ng/ml, UC = 2.82 ng/ml, CP = 7.08 ng/ml, IA = 1.56 ng/ml; Fig. 1D).

Taken together these results identify a protocol based on peptide affinity that effectively captures purified EVs and *BRAF*^V600E^ -bearing DNA fragments spiked into healthy donor plasma.

### Peptide affinity isolates both EVs and cell free DNA to enrich for mutant DNA

**Figure 2:**
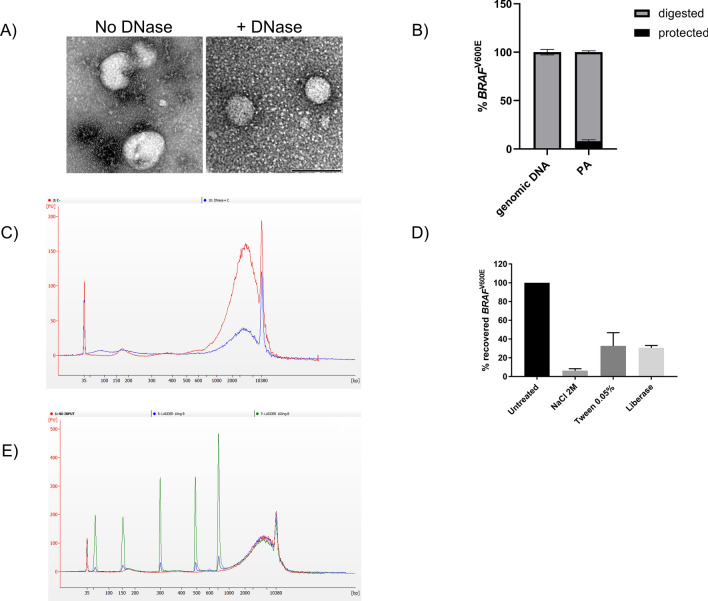
PA efficiently captures both EV surface bound DNA and cell-free DNA. (**A**) Trasmission electron microscopy (TEM) analysis of EV pellets obtained with PA isolation prior and after Dnase I digestion. DNase-sensitive aggregates (negative staining) were observed in close proximity to the EV surface and in EV-free areas. Images are representative of three independent experiments. Size bar (black) is 100 nm. (**B**) Quantitation of protected mutant DNA in PA pellet. DNA was extracted and used to detect mutant BRAF by AS-QPCR. Genomic DNA from a commercial supplier was used as internal control. Data were expressed as percentage of digested or protected mutation and representative of three independent experiments. (**C**) Electropherogram analysis of DNA obtained before (red line) and after Dnase I digestion (blue line) of PA-derived pellet. Graph is representative of three independent experiments. (**D**) Recovery of *BRAF*^V600E^-positive extracellular vescles (EVs) from healthy donor plasma samples after peptide affinity (PA) isolation in the presence of NaCl (2 M), Tween-20 (0.05%) or enzymatic digestion with liberase. Following DNA extraction, mutant *BRAF* was detected by allele-specific quantitative PCR (AS-QPCR). Results are representative of three independent experiments. (**E**) Electropherogram analysis of 10 and 100 ng of ladder DNA spiked into healthy donor plasma (blue and green line respectively; no input sample red line) and isolated by PA. Fragment size were 50 bp, 150 bp, 300 bp, 500 bp, 766 bp. Electropherogram is representative of three independent experiments.

To better understand the topology of the association of DNA to EVs, PA pellets obtained from EV-spiked plasma samples were digested with DNase I. Transmission electron microscopy (TEM) analysis showed that most of the isolated vesicles are in the size range of exosomes (diameter < 100 nm) and revealed the presence of DNase-sensitive molecular aggregates in close proximity to vesicle surface as well as in EV-free areas (negative staining; Fig. 2A). Consistent with this observation, only 8% of PA-captured mutation was found to be protected from enzymatic digestion, indicating that the mutation is mostly carried by unprotected DNA in this spike-in model (Fig. 2B). As control readout, spiked-in *KRAS*^G13D^ mutation was completely digested in these samples indicating optimal enzymatic activity on unprotected DNA (Suppl. Fig. 2). Electropherogram analysis of DNA from PA pellets showed the presence of DNA fragments ranging from 160 bp to several kilobases (Fig. 2C). While the 160-bp DNA peak has been previously identified as cell free circulating DNA in plasma^26^, 1 Kb or larger DNA fragments have only recently been associated to EVs^20,21^. In accordance with these reports, only these large DNA fragments were found to be partially protected from nuclease digestion (Fig. 2C).

Next, we investigated whether the peptide interacts with DNA-EV complexes through electrostatic forces, hydrophobic (lipid) or protein-mediated interactions. Strikingly, high salt concentration (2 N) almost completely abolished mutation recovery indicating that charge-based affinity is the primary mechanism for DNA-peptide interaction (6% recovery; Fig. 2D). Treatment with detergent Tween-20 or proteolytic digestion with liberase mix did not affect *BRAF*^V600E^ recovery as much as high salt (33% and 30% recovery respectively; Fig. 2D). Consistent with this mode of interaction, PA efficiently captured as little as 10 ng of synthetic DNA fragments, ranging between 50 and 750 bp, from 1 ml of plasma (Fig. 2E). The peptide’s ability to capture DNA fragments < 160 bp is an attractive feature for enriching genetic variants in plasma where tumor derived DNA has been previously reported to be shorter than somatic DNA^26^.

**Figure 3:**
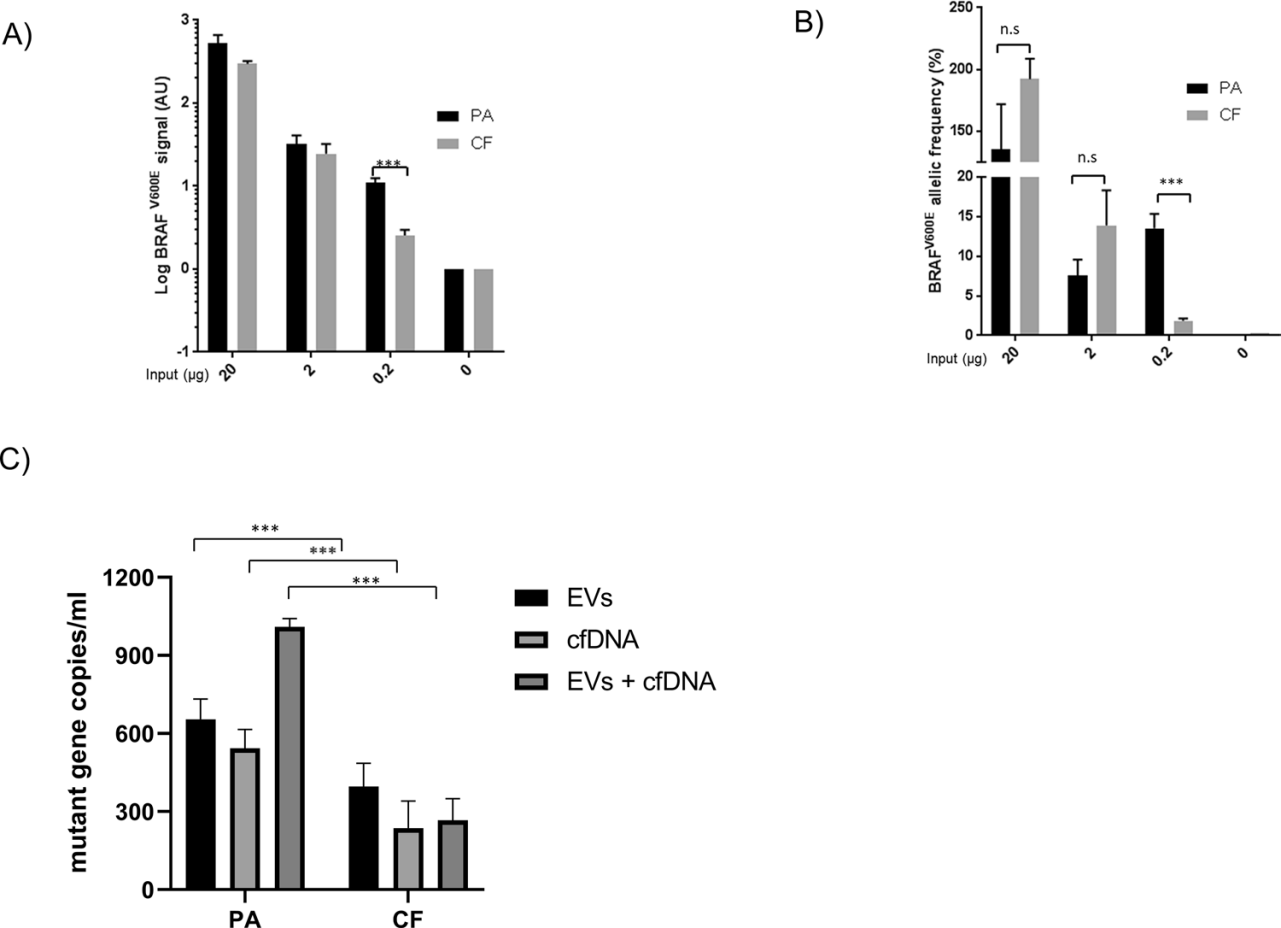
Peptide-based affinity isolation improves the detection of mutant DNA from ctDNA and EVs in low-copy number samples. (**A**) Recovery of decreasing amounts of mutation-positive extracellular vesicles (EVs) from healthy donor (HD) plasma samples with peptide affinity (PA) isolation and cell-free circulating nucleic acid isolation protocol (CF). Following DNA extraction, *BRAF*^V600E^ gene copies were detected by allele-specific quantitative PCR (AS-QPCR) and expressed as arbitrary units (AU) on a logarithmic scale. (**B**) *BRAF*^V600E^ allelic frequency was calculated after quantifying *BRAF*^WT^ gene copies by AS-QPCR and expressed as % on a logarithmic scale. Data are representative of three independent experiments. (**C**) Recovery of mutant DNA from both *BRAF*^V600E^-positive EVs and *KRAS*^G12S^-positive cfDNA by PA and CF isolation. 2.5 µg of *BRAF*^V600E^-positive EVs and 10 nanograms of *KRAS*^G12S^-positive cfDNA were spiked into HD plasma and isolated by PA and CF isolation. Following DNA extraction, mutant copies were quantified by digital PCR (dPCR). *BRAF*^V600E^ gene copies and *KRAS*^G12S^ gene copies were considered as total mutant copies in samples where cfDNA and EVs were co-spiked in the same plasma. Results are representative of three independent experiments.

Since PA recovered *BRAF*^V600E^ mutation from both EV-surface bound DNA and cfDNA, it was next compared to the current state-of-art protocol for cfDNA-based liquid biopsies (CF; Fig. 3A,B). To do so, decreasing amounts of *BRAF*^V600E^—positive EVs were spiked into healthy donor plasma and isolated with both methods. PA significantly isolated more *BRAF*^V600E^ mutation than CF only from the sample with the lowest amount of spiked-in EVs (0.2 μg, *P* value = 0.0007, Fig. 3A). No significant differences were observed in other samples, (20 μg, *P* value = 0.053; 2 μg, *P* value = 0.32; Fig. 3A). Significant mutation enrichment was also limited to the 0.2-ug sample, though notable differences were observed in other samples perhaps due to extraction variability, while no mutation was detected in the healthy donor plasma without EV input (*P* value = 0.0004; Fig. 3B). To further confirm these data, mutation-bearing EVs and cfDNA were spiked alone and together into HD plasma and isolated by both methods. PA isolated more mutation than CF from both biological sources, suggesting that PA is a more efficient way to isolate both EVs and cfDNA than CF rather than an enrichment tool for a single biomarker source (*P* value < 0.0001 for each comparison, Fig. 3C).

Overall, these results confirm that PA and the current standard technology for liquid biopsy, CF, effectively isolate tumor derived variants from high mutation burden samples. However, these data also point out that PA affinity may more efficiently isolate clinically actionable mutations from plasma samples with low mutation burden by capturing both EVs and cfDNA.

### Isolation of both cfDNA and EV-associated DNA improves *BRAF* gene detection in clinical samples

**Figure 4:**
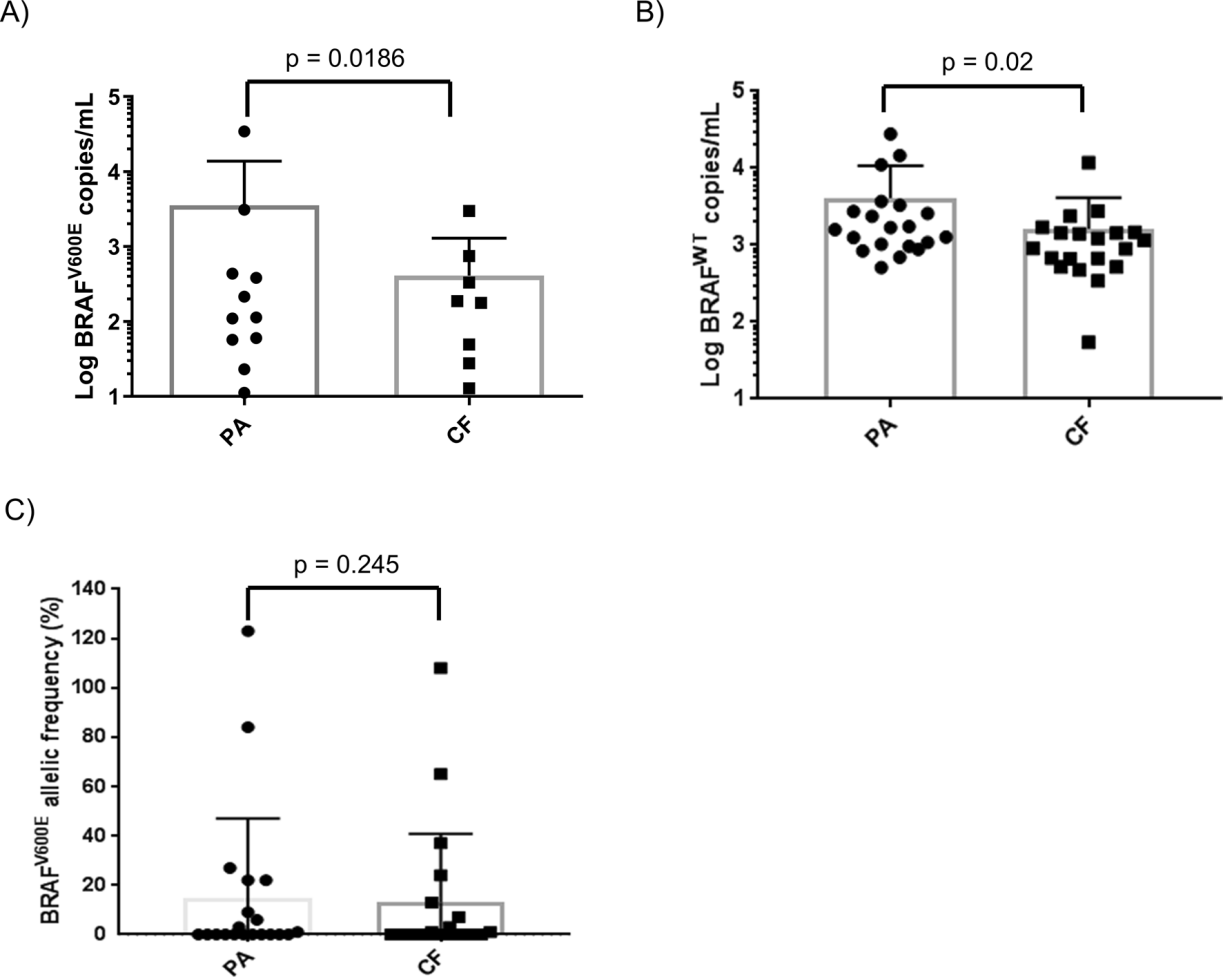
Peptide affinity-(PA) isolation captures more mutant and wild type *BRAF* gene copies than cell-free DNA protocol (CF) from plasma of *BRAF*^V600E^-positive metastatic melanoma (MM) patients. (**A**) *BRAF*^V600E^ copies or (**B**) *BRAF*^WT^ copies were isolated with PA or CF protocols from MM plasma sample of the mutant cohort and quantified by digital PCR (dPCR). (**C**) Mutation allelic frequency was calculated as the ratio between mutation and WT copies per ml of plasma and expressed as percentage (%).

To confirm the diagnostic benefit observed in spike-in models, a clinical study was set up comparing PA and CF as isolation methods for *BRAF* gene copies in patients’ plasma. Plasma samples were collected from twenty patients with *BRAF*^V600E^–positive tumors and thirty patients with wild type (WT) metastatic melanoma (MM) based on tissue biopsy examination (Suppl. Table 2). These plasma samples were collected at baseline or after 1 to 3-month treatment with BRAFi for *BRAF*^V600E^–positive patients or checkpoint inhibitors for WT patients (Suppl. Table 2 and 3). Five hundred microliters of plasma were processed with PA or CF and copies of *BRAF*^V600E^ and *BRAF*^WT^ were detected by digital PCR with LOD of 0.1% (Suppl. Fig. 3), concordant with the findings of Sanmamed et al. *BRAF*^V600E^ gene copies were detected in 11 PA- and 8 CF-processed plasma samples of the mutant cohort (PA sensitivity = 56%, CF sensitivity = 44%; Fig. 4A). Concordance rate between PA- and CF-processed samples was 80%. On average, PA isolated more mutant and WT copies than CF (Average *BRAF*^V600E^ gene copies/ml: PA = 1946.18; CF = 235.22; Average *BRAF*^WT^: PA = 4095.405, CF = 1624.36; Wilcoxon matched pairs ranked test *P* value *BRAF*^V600E^ = 0.0186; Wilcoxon matched pairs ranked test *P* value *BRAF*^WT^ = 0.002, Fig. 4A,B). *BRAF*^V600E^ allelic frequency of PA and CF-processed plasma samples was 15% and 13% respectively (Wilcoxon matched pairs ranked test *P* value = 0.25; Fig. 4C).

**Figure 5:**
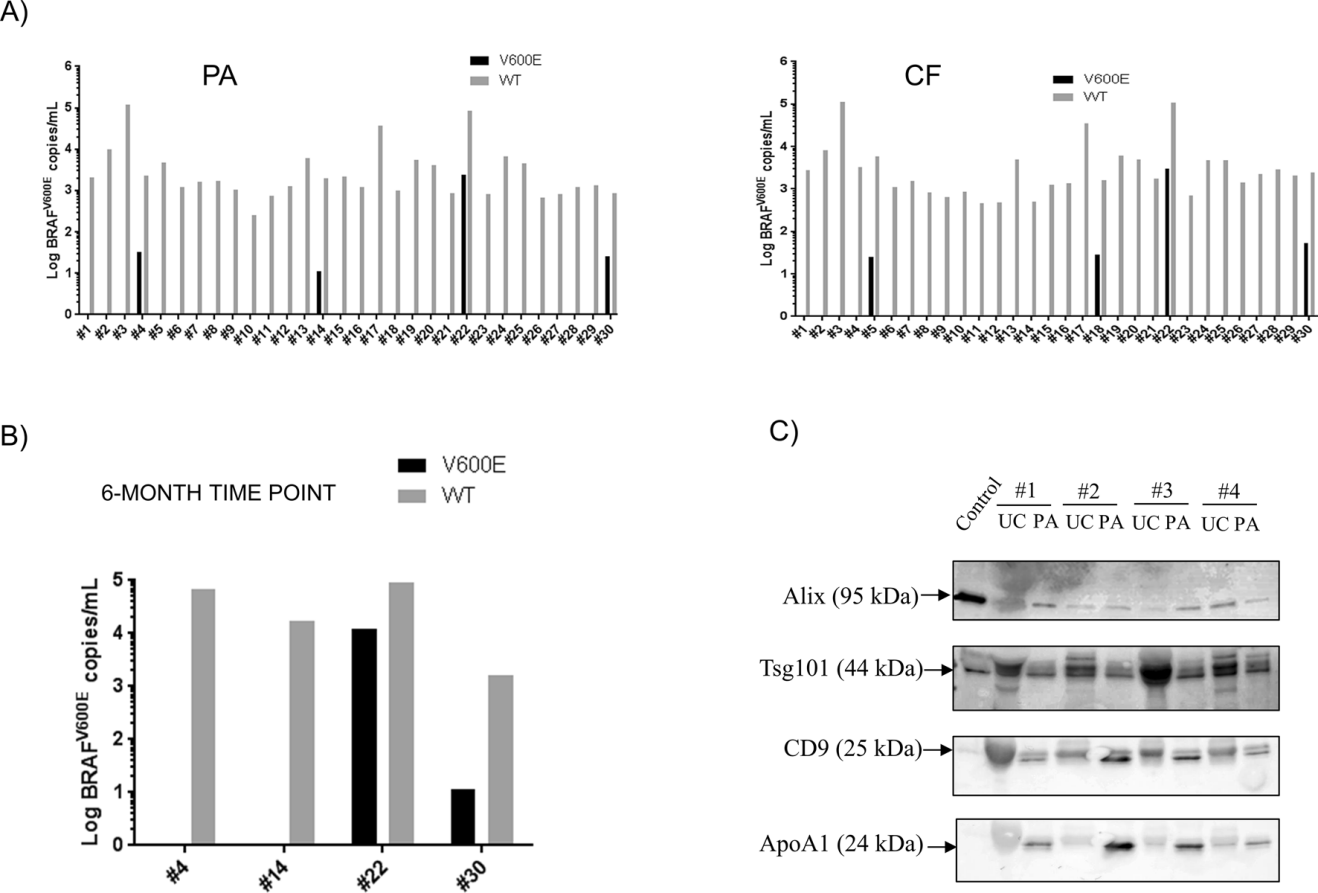
Detection of circulating *BRAF*^V600E^ gene copies from plasma of wild type (WT) metastatic melanoma (MM) patients. (**A**) *BRAF*^V600E/WT^ gene copies were isolated with PA or CF protocols from the plasma of WT cohort and quantified by digital PCR (dPCR). *BRAF* gene copies were expressed as gene copies per ml of plasma on a logarithmic scale. (**B**) Confirmatory study of the true positive status of plasma samples. Plasma samples from circulating *BRAF*^WT^-positive patients, previously tested as mutation positive in the PA-processed plasma, were collected 6 months after the initial time point and processed with PA protocol. *BRAF* gene status was determined by digital PCR and expressed as gene copies per ml of plasma on a logarithmic scale. (**C**) Western blot analysis on exosomal markers from plasma of metastatic melanoma patients. Total proteins were extracted from EV pellets from PA and UC and loaded on SDS-page, blotted onto a nitrocellulose membrane and stained with antibodies against exosomal markers Alix, TSg101, CD9 and non exosomal marker APOA1. Fifteen micrograms of purified exosomes ExoRef™ were used as control. Results are representative of three independent experiments.

*BRAF*^V600E^ copies were amplified in 4 PA- and CF-processed plasma samples of the WT cohort (specificity of both methods = 86.7%; Fig. 5A). Of these samples, two patients were found positive after both isolations and re-tested positive after 6 months (patient #22 and #30; Fig. 5A, B). All plasma samples from healthy controls were found free of this mutation (Suppl. Fig. 4). Moreover, western blot analysis of exosomal markers Alix, TSG101 and CD9 on plasma samples of four metastatic melanoma patients from the WT cohort confirmed that PA isolates EVs from these samples (Fig. 5C). Minimal variability was observed between samples stained with anti-Alix antibodies while TSG101 and CD9 expression were slightly more variable between isolation techniques and across patient’s samples (Fig. 5C; Suppl. Fig. 5B). Total protein staining with Ponceau Red and immunostaining with anti-APOA1 antibodies indicated that PA pellets contain substantially less proteins but more non exosomal APOA1 lipoprotein than UC pellets, suggesting that the peptide copurifies lipoprotein-rich particles such as HDL from plasma (Fig. 5C and Suppl. Fig. 5A).

**Figure 6:**
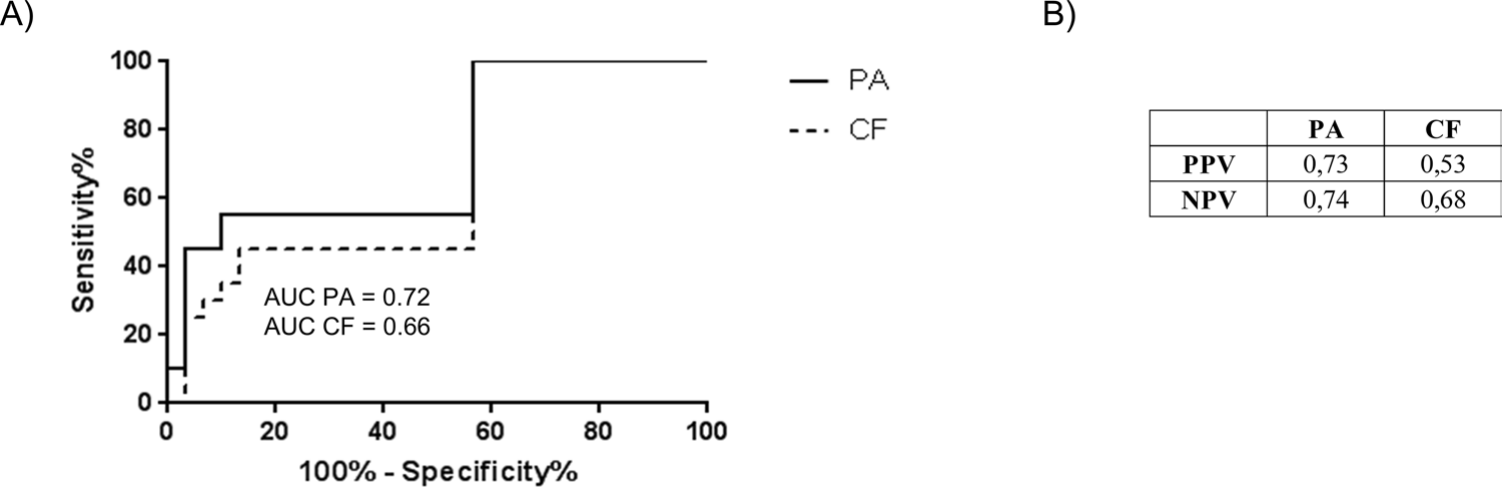
Receiver Operating Characteristic (ROC) analysis and positive/negative predictive values (PPV/NPV) of circulating *BRAF*^V600E^ from plasma of mutant and WT patients after PA and CF protocols.

ROC curve analysis of *BRAF*^V600E^ copy number from PA and CF isolations generated an area under the curve (AUC) of 0.72 and 0.66 respectively (Fig. 6A). Positive and negative predictive (PPV/NPV) values of PA were higher than CF (Fig. 6B; PA PPV = 0.73 NPV = 0.74; CF PPV = 0.53 NPV = 0.68). Taken together, these data highlight an incremental but significant improvement in the detection of *BRAF*^V600E^ gene copies using DNA isolated by PA isolation.

**Figure 7:**
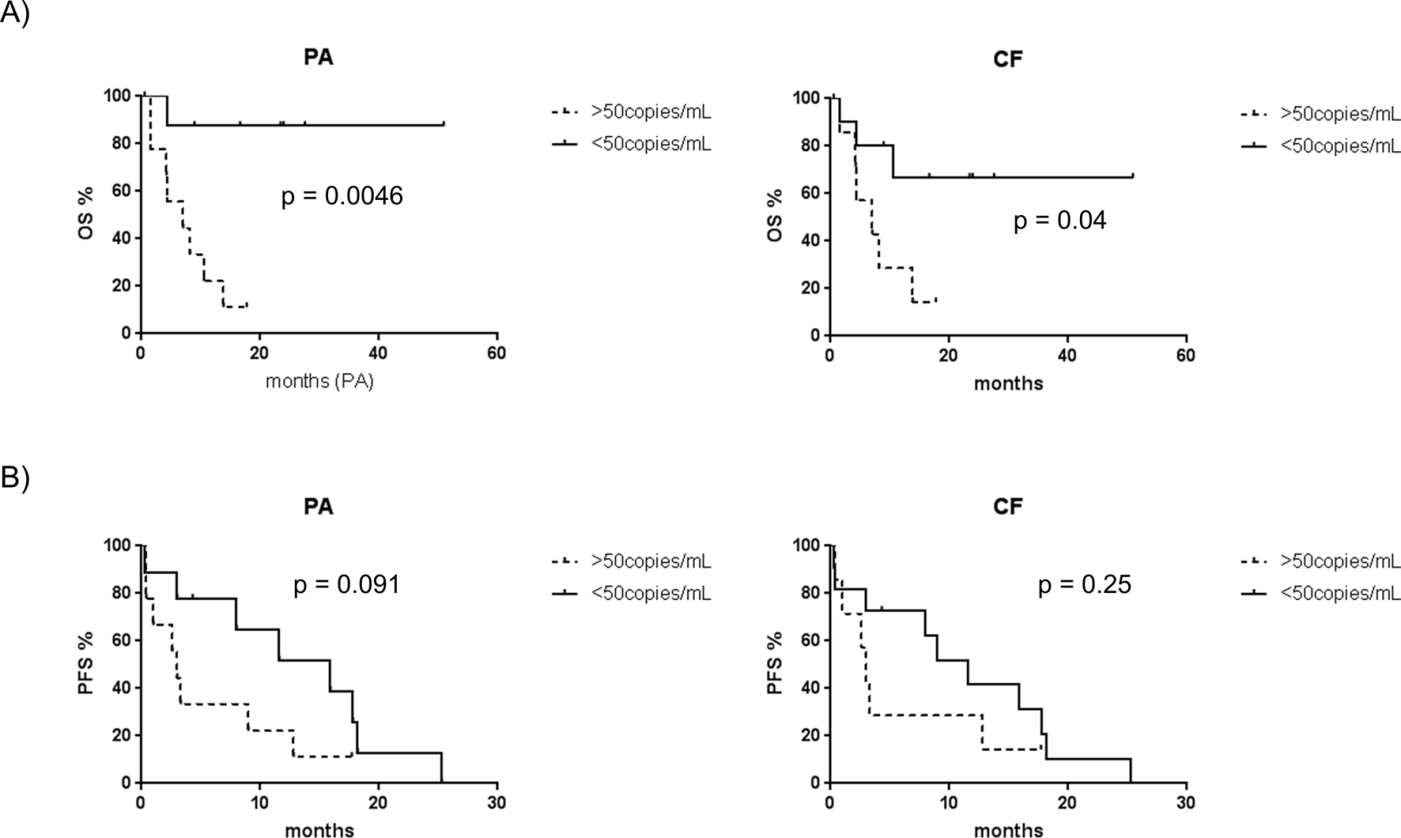
Prognostic value of circulating *BRAF*^V600E^. (**A**) Overall survival (OS) and (**B**) progression-free survival (PFS) curves were calculated for PA- and CF isolation protocols by setting a threshold of 50 mutant copies per ml of plasma. Log-rank test was used to determine the *P* value.

Since circulating levels of *BRAF*^V600E^ have been previously linked to poorer prognosis in MM^8^, *BRAF*^V600E^’s prognostic power was assessed after both isolation protocols. Overall survival (OS) and progression-free survival (PFS) of the mutant cohort were calculated by setting an arbitrary threshold of 50 mutant gene copies per ml of plasma (Suppl. Table 3). Patients with above-threshold mutant copies had median OS value of 7 months and PFS value of 3 months with both methods (Fig. 7A,B, Suppl. Table 3). Median PFS values of patients with below-threshold copy levels of *BRAF*^V600E^ were 15.9 and 11.6 months after PA and CF isolation respectively (Fig. 7B).

As expected, average PFS and OS were 18.2 months and 12.9 months in patients with partial or complete response to BRAF inhibitors as opposed to patients with stable or progressing disease (PFS 3.5 months; OS 1.8 months; Suppl. Table 3).

Log-rank test P values of OS and PFS curves were lower in PA- than CF-processed samples, confirming the value of circulating *BRAF*^V600E^ as negative prognostic biomarker in MM patients (Log-rank test, OS: *P* value_PA_ = 0.0046; *P* value_CF_ = 0.04; PFS: *P* value_PA_ = 0.091; *P* value_CF_ = 0.25; Fig. 7A,B).

**Figure 8:**
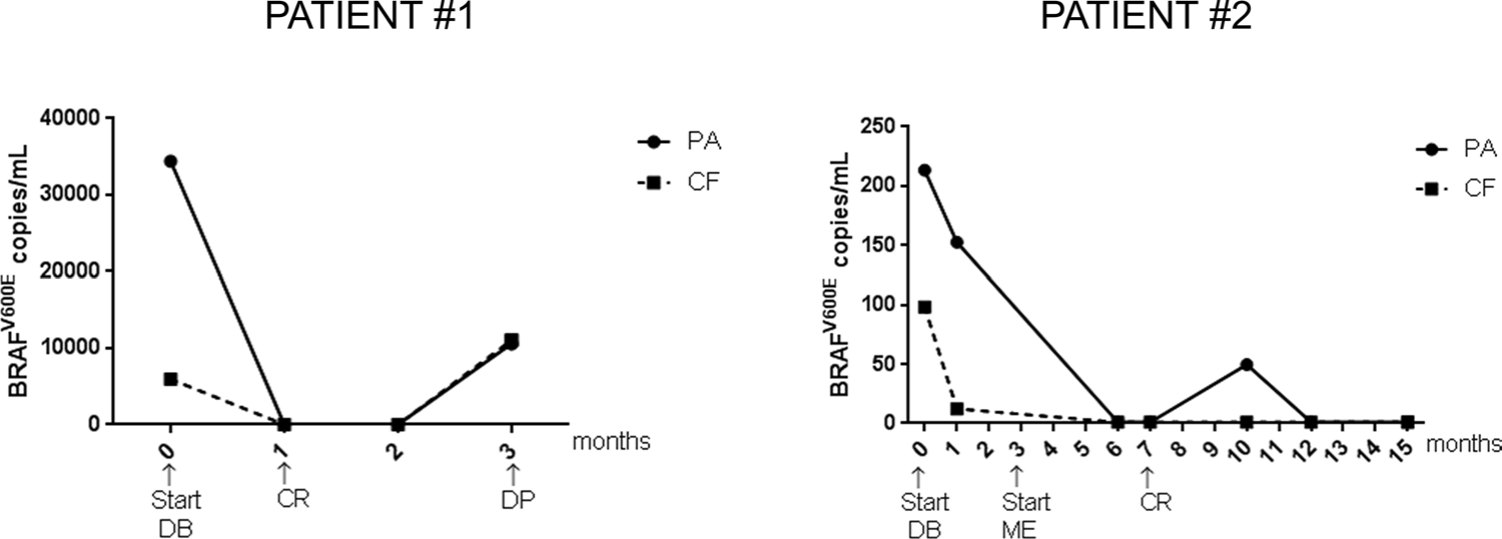
Monitoring *BRAF*^V600E^ copy levels during BRAF inhibitor (BRAFi) treatment. *BRAF*^V600E^ copies were isolated using PA or CF protocols from plasma of two melanoma patients before and during BRAFi treatement and detected by digital PCR. DB = Dabrafenib; ME = Mekinist; CR = complete response; DP = disease progression.

Finally, both sample-prep protocols were used for monitoring *BRAF*^V600E^ levels in the plasma of BRAF inhibitor-treated MM patients. Two MM patients were selected with high and low burden of circulating *BRAF*^V600E^ at the beginning of treatment (patient #1 PA = 13,419 gene copies/ml, CF = 5966 gene copies/ml; patient #2 PA = 213.45 gene copies/ml, CF = 98.21 gene copies/ml; Fig. 8). In these patients, treatment with Dabrafenib and/or Mekinist (DB, ME respectively) led to a clinical response (CR) within the first 7 months of treatment and correlated to diminishing levels of mutation in both PA and CF-processed plasma samples (Fig. 8). In patient #1, with high burden of circulating mutation at baseline, disease progression (DP) occurred within 3 months and was associated to rebounding levels of circulating *BRAF*^V600E^ and unfavorable prognosis (Fig. 8;^8^). In patient #2, with low tumor mutation burden, no clinical evidence of disease progression was observed at later time points, and mutant gene copies remained low or undetectable in plasma (49 copies/ml at 10-month post treatment; Fig. 8). Consistent with our data from spike-in samples (Fig. 3B), PA but not CF, significantly enriched for the mutation in this patient (patient #2; Suppl. Fig. 6). Taken together, these results suggest that DNA isolation based on peptide affinity to both EVs and cell free DNA may improve the detection of circulating *BRAF*^V600E^ in patients with low tumor mutation burden.

## Discussion and conclusions

While a growing body of evidence supports the use of EVs as promising carriers of tumor biomarkers, including BRAF^*V600E*^^14–19^, their clinical adoption has been slowed by the lack of isolation protocols that would obviate the need for ultracentrifugation (UC^25^). In this paper, we report an UC-free isolation protocol based on peptide-mediated affinity (PA) that efficiently isolates both extracellular-vesicle-(EV)-DNA and cfDNA from plasma. The protocol recovers twice as much *BRAF*^V600E^ mutation as UC in a spike-in system, and with a much lower turnaround time of 2 h and hands-on time of 10–15 min. PA isolates large amounts of EVs with respect to antibody-based affinity but comparably to more generic EV-isolation protocols such as UC and PEG-based precipitation. This is not surprising as PA may isolate EVs that do not express tetraspanins on their surface. Furthermore, TEM and DNase protection assay revealed that more than 90% of mutant DNA isolated by PA is outside of the EVs, associated with the outer surface of the EV membrane or independently co-purified within protein aggregates. This result is consistent with the existence of a recently proposed mechanisms for active co-secretion of nucleosomes and exosomes from multivesicular bodies of cancer cells^27^. Furthermore, nucleosomes and EV-rich complexes protect DNA fragments larger than 1 Kb as previously observed^20,21^. Dwelling deeper into the chemical nature of peptide-DNA-EV interaction, we found that increasing ionic strength with salt abolishes the formation of the complex. On the other hand, the peptide forms complexes with short DNA fragments, ranging from 50 to 750 bp, without direct association to EVs. Spike-in experiments using both mutant-bearing EVs and cfDNA in healthy donor plasma confirmed that PA efficiently isolates both sources from plasma, especially when the concentration of biomarkers is low. In comparison to UC, however, PA pellets contain less plasma proteins but more lipoprotein APO-A1, indicating that PA isolates non exosomal nanoparticles, such as HDL, from plasma of melanoma patients. RNA/DNA-lipoprotein macro-complexes are released from both living and dying cells and they are, besides EVs and free circulating nucleic acids, important carriers of circulating nucleic acids in plasma^28,29^. While restricted to a limited sample set, these data suggest that the peptide’s ability to isolate more efficiently multiple mutation sources but less plasma proteins than generic isolation methods may be at the basis of the improved mutation detection. Further experiments comparing PA to other isolation methods on a larger sample set are warranted to confirm this hypothesis.

Compared to the state-of art of isolation for cell-free circulating nucleic acids (CF), PA isolates more mutant and wild type *BRAF* gene copies but, consistent with spike-in experiments, isolated more mutant alleles in plasma samples of patients with low mutation burden (Fig. 8 and Suppl. Table 3). While PA protocol isolates more mutant copies than CF, its diagnostic sensitivity was still low compared to previous studies (56%^30,31^). Two reasons may explain this performance: (A) the use of 500 µl of plasma instead of 1–2 ml of more as previously reported^31^; (B) the mutant cohort included patients undergoing 1–3 months of BRAFi treatment as shown in Supplementary Table 3. Consequently, responders had undetectable levels of circulating mutation, affecting sensitivity of both protocols. In fact, restricting the analysis to untreated patients increased the sensitivity to 73%. Specificity of both protocols, obtained using tissue biopsy results as reference of diagnostic performance^32^, was 87% as mutation was also detected in four out of thirty plasma samples of the WT cohort. As for the mutant cohort, diagnostic specificity of the test may have been affected by treatment as all WT patients were treated for 1 to 3 months with checkpoint inhibitors at the time of sampling (Suppl. Table 2). No mutation was detected in healthy donor plasma (Suppl. Fig. 4).

In this clinical study PA isolation improved both PPV and NPV of the test in comparison to CF, however, the definition of false positives and true negatives in the WT cohort may be blurred by the fact that later re-testing confirmed the true positive status of two samples (Fig. 5B), suggesting that liquid biopsy may indeed capture molecular events that are missed by conventional biopsy due to tumor heterogeneity^32^. Clinical studies with confirmatory re-biopsies are warranted to better distinguishing false positives (and true negatives) from true positive samples within both cohorts.

Consistent with previous reports^8,33^, *BRAF*^V600E^ copy levels above 50 copies/ml were found to correlate to poorer overall survival of MM patients after both PA and CF protocols but the enhanced sensitivity of the PA protocol improved the mutation’s prognostic value.

As expected, PFS and OS were higher in patients showing partial or complete response to BRAF inhibitors (18.2 months and OS of 12.9 months respectively) as opposed to patients with stable or progressing disease (PFS 3.5 months; OS 1.8 months). Interestingly, PA enabled the detection of three more mutant positive samples than CF, two of which under complete clinical response and mutant copy number below 50 copies/ml of plasma.

In our biomarker monitoring study, both protocols showed that *BRAF*^V600E^ copy levels drop to undetectable after BRAFi treatment but quickly bounce back as disease progresses. As expected, PA isolation increases mutant allele frequency only in plasma from the patient with lowest mutation burden while keeping high sensitivity in less challenging samples. Taken together, our data suggest that PA isolation protocol may provide a diagnostic and prognostic benefit especially in patients with naturally occurring or therapeutically-induced low levels of mutation. Although not directly demonstrated by this study, the increased sensitivity of the PA protocol is likely to be due to the affinity capture of tumor-derived EVs, which in MM plasma are a small but significant EV fraction associated to tumor-derived *BRAF*^V600E^ mutation^34^. While our in vitro experiments confirm that exosomes do not carry significant amount of DNA internally, other larger vesicles released in pathological conditions, such as oncosomes, may incorporate DNA^35^ partially explaining the improved recovery of mutant DNA in patient’s plasma. Furthermore, studies to identify mutation-rich EV fractions in plasma or other body fluids^36^ and to improve the efficiency and stringency of the affinity isolation with novel tumor-specific binding agents (eg. antibodies or aptamers) are warranted^37–40^.

In perspective, we envision the clinical use of protocols that enrich for tumor-derived EVs to improve the detection of actionable mutations beyond *BRAF*^V600E^ (eg. mutation associated to resistance mechanisms to BRAFi) from EV-DNA. Further clinical validation of the proposed protocol will demonstrate whether co-isolation of EVs and cfDNA improves sensitivity of the current state-of-art for liquid biopsy to support stratification and monitoring of MM patients when re-biopsy of tumor tissue is not a viable option.

## Materials and methods

All materials and methods were performed in accordance with the relevant guidelines and regulations (more details in the following sections).

### Cell lines and exosome purification from cell supernatant

The BRAF V600E–homozygous cell line (A375) from malignant melanoma was obtained from American Type Culture Collection (Cat Num. CRL-1619, ATCC). Cells were supplemented with Dulbecco’s Eagle Modified’s Medium (DMEM), 10% heat inactivated bovine serum (FBS), 1% glutamine, 1% penicillin and streptomycin (Gibco) and incubated at 37 °C in a 5% CO_2_ humidified incubator. After a 72-h incubation in media with FBS, cell supernatant was collected and filtered with a 0.22 µM filter (Thermo Fisher Scientific) to eliminate cell debris. A375 exosomes were isolated by ultracentrifugation as previously described^23^. Total protein content was determined by direct detect infrared spectrometry (Millipore).

### Patient’s characteristics and blood processing

A total of 50 patients with unresectable metastatic melanoma stage IIIC–IV were included in this study from January 2011 until December 2014 and followed-up until December 2016. The study was approved by Ethical Committee of the Azienda Ospedaliera Universitaria Le Molinette, Torino, Italy and plasma samples were collected from patients after informed consent (Comitato Etico Interaziendale Titolare A/2.10 del 03-07-2014 Prot. N. 0068188). All patients underwent tumor biopsy, which revealed that 20 of patients’ tumors (40%) were *BRAF*^V600E^-positive and 30 (60%) *BRAF*^WT^-positive. The clinical characteristics of patients are summarized in Supplementary Table 2. There was a female prevalence with a median age of 67 years. The primary sites were cutaneous (78%); mucosal or uveal (12%; all *BRAF*^WT^) and unknown (10%). Most patients had stage IV, predominantly M1c (76%); only 2 patients had unresectable stage IIIc (all BRAF WT). Patients with wild type *BRAF* gene disease were treated at the time of sampling by ipilimumab (n = 23) or anti-PD1 nivolumab (n = 8). *BRAF*^V600E^-positive patients underwent treatment with targeted therapies. Only one *BRAF*^V600E^-mutant patient underwent first line treatment with anti-PD1 nivolumab. Tumour response was evaluated on the basis of physical examination and radiological procedures using Response Evaluation Criteria in Solid Tumours (RECIST) version 1.1. The best response was defined as the best objective response (complete response, partial response, stable disease, progressive disease) as assessed between the first day of treatment and last follow-up or death.

Tumor biopsy and plasma sampling were performed in each case before initiation of the treatment recorded in the study. For mutation monitoring during therapy, plasma was collected every month for a maximum of 15 months. To obtain plasma for the study, 10 ml of blood were collected in K2-EDTA Vacutainer® tubes (Cat Num. 366643; Becton Dickinson) using a butterfly system with needle gauge of 21 (Cat Num. 367281; Becton Dickinson). Blood tubes were then mixed 8 times and processed with 4 h from harvest by centrifugation at 1500*g* for 15 min at RT. Plasma was aliquoted in labeled cryotubes and stored at − 80 °C prior to use.

### Isolation of EV from plasma

Before EV isolation all plasma samples were centrifuged at 1200*g* for 20 min at 10° Celsius to eliminate red blood cells and cellular debris.

EV isolation on plasma samples by ultracentrifugation was performed as previously described^21^. EV isolation by chemical precipitation (CP), peptide affinity (PA) and immunoaffinity (IA) was performed according to the manufacturer’s instructions (ExoPrep, Cat Num. HBM-EXP-B5 HansaBioMed Life Sciences OU; peptide based isolation step of SeleCTEV™ Low Input DNA Enrichment Kit, Exosomics SpA; CD9/CD63-antibody-coated-latex beads, Cat Num. HBM-BOLF-CC/10-1, HansaBioMed Life Sciences OU). In the case of PA protocol, the isolation buffer was modified by adding 2 N NaCl (Cat Num. S7653; Sigma-Aldrich), Tween-20 (Cat Num. P9416; Sigma-Aldrich) at the final concentration of 0.05% or liberase™ mix used according to the manufacturer’s instructions (Cat Num. 5401119001; Sigma Aldrich) to investigate the nature of interactions established between peptide and DNA-EV complexes.

### Isolation of cfDNA and synthetic DNA from plasma

Cell-free DNA was isolated using the QIAamp Circulating Nucleic Acid kit according to the manufacturer’s instructions (Cat Num. 55114, Qiagen).

For spike-in experiments, 10–100 µl of ladder DNA were purchased from a commercial vendor (Quick Load PCR Market N047S; New England Biolabs) and spiked into 1 ml of healthy donor plasma. DNA was recovered using PA-based kit according to the manufacturer’s instructions (Isolation step of SeleCTEV™ Low Input DNA Enrichment Kit, Exosomics SpA). For mutation detection experiments, standard genomic DNA bearing the KRAS G12S mutation (Cat Num. HD288, Horizon Discovery) was fragmented using a M220-focused ultrasonicator following the manufacturer’s instructions (Covaris) to obtain DNA fragments with average size of 228 bp.

### DNase I protection assay

EV pellets and genomic DNA were resuspended in a digestion mix containing 80 μL of PBS, 10 µl of DNase I and 10 µl of Reaction Buffer and incubated for 15′ at RT (Cat. Num AMPD1, Sigma-Aldrich). After incubation, the DNase activity was blocked by adding 50 mM EDTA and samples were heated at 70 °C for 10 min to inactivate the enzyme before further analysis.

### DNA purification and electropherogram analysis

Extracellular vesicle-associated DNA (EV-DNA) was extracted from each isolation pellet using the SeleCTEV™ DNA sample-prep kit for circulating DNA extraction and following its instructions (DNA purification step from SeleCTEV™ Low Input DNA Enrichment Kit; Exosomics SpA). Cell-free DNA was extracted using the QIAamp Circulating Free Nucleic Acid kit according to the manufacturer’s instructions (Cat Num. 55114, Qiagen). To determine DNA fragment size, electropherogram analysis was performed using the High Sensitivity DNA kit on a 2100 Bioanalyzer instrument according to the manufacturer’s instructions (Cat Num. 5067-4626, Agilent Technologies).

### Allele-specific PCR

Allele-specific PCR was a modified version of the allele-specific locked nucleic acid PCR published by Morandi et al.^41^. Briefly, each qPCR reaction included 10 µl of DNA eluate or 10 ng of genomic DNA as positive control (Cat Num. HD238, Horizon Discovery), 1X SsoAdvanced Universal Probes Mastermix (Cat Num. 172-5285, Bio-Rad, US), 0.625 µl of primers (10 µM) and 0.3125 µl of fluorescent probe (10 µM) in a total volume of 25 µl. After careful mixing, each reaction was loaded in triplicate on a 96-well PCR plate and the following qPCR program was launched: 95 °C for 3′, 40 cycles at 95 °C for 5″ and 60 °C for 30″. To calculate the % of recovered *BRAF*^V600E^ gene the following formula was used: = [2^(Ct input – Ct isolation)]%

### Western blot analysis

Pellet samples were resuspended in an appropriate volume of Protein Loading Buffer (Cat Num. 00193861, Lonza BV), equal sample volumes were separated by SDS-PAGE on precast gels (Cat Num. 4561095, Bio-Rad US). In Fig. 5C, fifteen micrograms of purified exosomes ExoRef™ (Exosomis SpA) were used as control sample. Proteins were transferred onto a nitrocellulose membrane (Cat Num. 15249794, GE Healthcare). Western blotting was run with primary antibodies against Alix (Cat Num. sc-271975, Santa Cruz), Tsg101 (Cat. Num. ab30871, Abcam), CD9 (Cat Num. 555370, BD Pharmigen), GAPDH (Cat Num. 25778, Santa Cruz), APO-A1 (Cat Num. ab17278, Abcam) and HRP-conjugated mouse or rabbit secondary antibody (Cat Num. P0260, Cat. Num. P0448; Dako). Mild stripping protocol from Abcam (Abcam https://www.abcam.com/ps/pdf/protocols/stripping%20for%20reprobing.pdf) was applied to strip to re-probe the same membrane with different antibodies. Chemiluminescence was produced using the SuperSignal™ West Femto Maximum Sensitivity Substrate (Cat Num. 34095, Thermo Fisher Scientific).

### Transmission electron microscopy (TEM)

Pellet samples, obtained after PA isolation of spiked-in plasma samples, were resuspended in 80 µl of PBS and digested with (or without) DNase 1, as previously described. Later, a 3-μl drop of sample was placed on a 300 mesh formvar coated copper grid for 2 min. Blotted the excess with filter paper, the grid was negatively stained with 1% aqueous Uranyl Acetate for 30 s and analyzed using a FEI Tecnai G2 Spirit TEM operating at 120 kV equipped with an EMSIS Veleta CCD camera.

### Digital PCR (dPCR)

Digital PCR experiment set up was performed according to the Digital MIQE Guidelines^37^.

Amplification was carried out in 15 µl volume using the QuantStudio 3D Digital PCR System Platform (Thermo Fisher Scientific, Carlsbad, CA, USA). PCR reaction mix contained 8 µl of 2X QuantStudio 3D Digital PCR Master Mix (Thermo Fisher Scientific), 0.4 µl of 40X TaqMan-MGB-FAM-probe assay (BRAF assay ID: BRAF_476_mu; KRAS G12S Assay ID: Hs000000048; KRAS G13D assay ID: Hs000000056_rm; Thermo Fisher) 1.1 µl of diluted DNA (50 ng/µl) and 6.5 µl of nuclease-free water. Serial dilutions of genomic DNA standards (Cat Num. HD238, Horizon Discovery) and of negative controls with DNA-free water were used to estimate the limit of detection. (Suppl. Fig. 3).

To amplify the target genes, 15 µl of the reaction mix were loaded onto a QuantStudio 3D Digital PCR 20 K Chip (Thermo Fisher Scientific) and run following this PCR program: 95 °C for 8′, 40 cycles at 95 °C for 15″ and 60 °C for 1′, followed by a final extension step at 60 °C for 2′. Raw data were analyzed with QuantStudio 3D Analysis Suite Cloud Software to calculate gene copies per ml.

### Statistical analyses

Paired parametric *t* test analysis was used to compare isolation methods from the same EV spike-in sample. The Wilcoxon signed-ranked test was used to compare PA and CF isolations from the same patient’s plasma sample. Progression-free survival and overall survival (OS) were measured from the start of BRAFi treatment to the disease progression, death, or last follow-up using the Kaplan–Meier method and compared by log-rank test.

## Supplementary information

Supplementary Information.
