# Supplementary material for: Isolation of extracellular vesicles improves the detection of mutant DNA from plasma of metastatic melanoma patients
Source: Sci Rep. 2020 Sep 25;10:15745. doi: 10.1038/s41598-020-72834-6 (PMC7519075; doi:10.1038/s41598-020-72834-6)
Supplement: Supplementary file 1 — Supplementary Information. [file 41598_2020_72834_MOESM1_ESM.pdf]

Supplementary Information

Title

Isolation of extracellular vesicles improves the detection of mutant DNA from plasma of metastatic melanoma patients

Davide Zocco[1*], Simona Bernardi[4,5], Mauro Novelli[2,3], Chiara Astrua[2,3], Paolo Fava[2,3], Natasa Zarovni[1] Francesco M Carpi[1], Laura Bianciardi[1], Ottavia Malavenda[2,3], Pietro Quaglino[2,3], Chiara Foroni[4,5] Domenico Russo[4,5], Antonio Chiesi[1], Maria Teresa Fierro[2,3]

EV Isolation	Technology	Commercial providers	Advantages	Barriers to clinical adoption	References
By physical properties (size, density, charge)	Ultracentrifugation	Several equipment manufacturers	Gold-standard protocol for EV isolation	Expensive equipment; cumbersome, lenghty protocol	24,31
	Sucrose/ioxidanol gradient fractionation	Several equipment and reagent manufacturers	Highest EV purity	Expensive equipment; cumbersome, lenghty protocol, not suitable for body fluids	31, 40
	Field flow fractionation	None	High purity and yield	Expensive equipment; top-bench FFF devices not available	45
	Size exclusion chromatography	qEV (Izon Science)<br>Pure-EV (HansaBioMed OU)	High purity; ease-of-use	Samples require further concentration steps for EV purification	46
By reduced solubility (chemical precipitation)	Polyethylen glycol-(PEG)-based solution	ExoQuick (SBI)<br>Total Exosome Isolation reagent (Thermo Fisher)<br>miRcury Exosome Isolation kit (Exiqon)<br>Exosome Purification Kit (Norgen)<br>PureEXO (101BIO)<br>EXO-Prep (HansaBioMed OU)	Ease-of use, tested on several types of body fluids; fast turnaround time	Low purity	44,47
	Salting-out	None	Ease of use, inexpensive	Not tested on body bluids	48
By affinity to EV-markers	Peptide-based capture	ME-kit (NEP)<br>SeleCTEV-sample Prep (Exosomics Spa)	Ease-of use, tested on several types of body fluids, enrichment of tumor-derived EVs; fast turnaround time	None	49
	Heparin-based capture	None	Ease of use, fast TAT	None	50
	Antibody-based capture	ExoCAP kit (JSR)<br>Exosome Streptavidin Isolation/detection (Thermo Fisher)<br>Exosome Immunocapture beads (HansaBioMed OU)	Ease-of use, tested on several types of body fluids, enrichment of tumor-derived EVs; fast turnaround time	None	30

Supplementary Table 1: Methods for isolation of extracellular vesicles

Title

Isolation of extracellular vesicles improves the detection of mutant DNA from plasma of metastatic melanoma patients

Davide Zocco[1*], Simona Bernardi[4,5], Mauro Novelli[2,3], Chiara Astrua[2,3], Paolo Fava[2,3], Natasa Zarovni[1] Francesco M Carpi[1], Laura Bianciardi[1], Ottavia Malavenda[2,3], Pietro Quaglino[2,3], Chiara Foroni[4,5] Domenico Russo[4,5], Antonio Chiesi[1], Maria Teresa Fierro[2,3]

Patient baseline characteristics (n=50)		
**Variable**	**Overall**	**Treatment**
**Age (years)**	67	17D, 3V, 23I, 7N
**Sex**		
Male	21 (42)	7D/1V/9I/4N
Female	29 (58)	10D/2V/14I/3N
**Stage**		
Unresectable IIIc	2 (4)	1I/1N
Iva	8 (16)	5D/3I
IVb	2 (4)	2I
IVc	38 (76)	12D/3V/17I/6N
**Primary site**		
Cutaneous	33 (66)	14D/2V/14I/3N
Mucosal	4 (8)	4I
Uveal	2 (4)	2N
Acral	6 (12)	2D/4I
Unknown	5 (10)	1D/1V/1I/2N
Data are mean (%) D, dabrafenib; V, vemurafenib; I, ipilimumab; N, nivolumab		

Supplementary Table 2: Patient baseline characteristics

Title

Isolation of extracellular vesicles improves the detection of mutant DNA from plasma of metastatic melanoma patients

Davide Zocco[1*], Simona Bernardi[4,5], Mauro Novelli[2,3], Chiara Astrua[2,3], Paolo Fava[2,3], Natasa Zarovni[1] Francesco M Carpi[1], Laura Bianciardi[1], Ottavia Malavenda[2,3], Pietro Quaglino[2,3], Chiara Foroni[4,5] Domenico Russo[4,5], Antonio Chiesi[1], Maria Teresa Fierro[2,3]

BRAF V600E-positive patients characteristics (n=20)						
Patient	BOR	Treatment	OS (months)	PFS (months)	BRAF^{V600E} copies/ml; PA	BRAF^{V600E} copies/ml; CF
#1	CR	D	27.5	15.9	22.95	0.00
#2	CR	D	23.9	17.8	11.16	0.00
#3	CR	D	50.9	25.3	0.00	0.00
#4	CR	D	10.5	9.0	109.65	27.68
#5	CR	N	N/A	N/A	0.00	0.00
#6	PD	V	0.5	0.3	56.85	12.83
#7	PD	D	4.3	3.0	0.00	0.00
#8	PD	D	1.5	0.4	113.25	0.00
#9	PD	D	4.2	2.6	380.40	186.47
#10	PD	D	4.3	3.0	0.00	0.00
#11	PD	V	1.5	0.4	3103.80	747.39
#12	PR	D	13.8	12.8	433.05	330.92
#13	PR	D	16.6	11.6	0.00	0.00
#14	PR	D	23.4	18.2	60.15	176.51
#15	PR	D	8.9	8.0	0.00	0.00
#16	PR	D	7.0	1.0	0.00	0.00
#17	PR	D	4.3	4.3	0.00	0.00
#18	PR	D	17.7	17.7	213.45	49.11
#19	SD	D	8.2	3.3	34419.00	2983.50
#20	PD	V	N/A	N/A	0	0
CR, complete response; PR, partial response; SD, stable disease; PD, disease progression						
D, dabrafenib; V, vemurafenib; N, nivolumab						

Supplementary Table 3: BRAF V600E-positive patients characteristics

Title

Isolation of extracellular vesicles improves the detection of mutant DNA from plasma of metastatic melanoma patients

Davide Zocco[1*], Simona Bernardi[4,5], Mauro Novelli[2,3], Chiara Astrua[2,3], Paolo Fava[2,3], Natasa Zarovni[1] Francesco M Carpi[1], Laura Bianciardi[1], Ottavia Malavenda[2,3], Pietro Quaglino[2,3], Chiara Foroni[4,5] Domenico Russo[4,5], Antonio Chiesi[1], Maria Teresa Fierro[2,3]

Supplementary Fig. 1

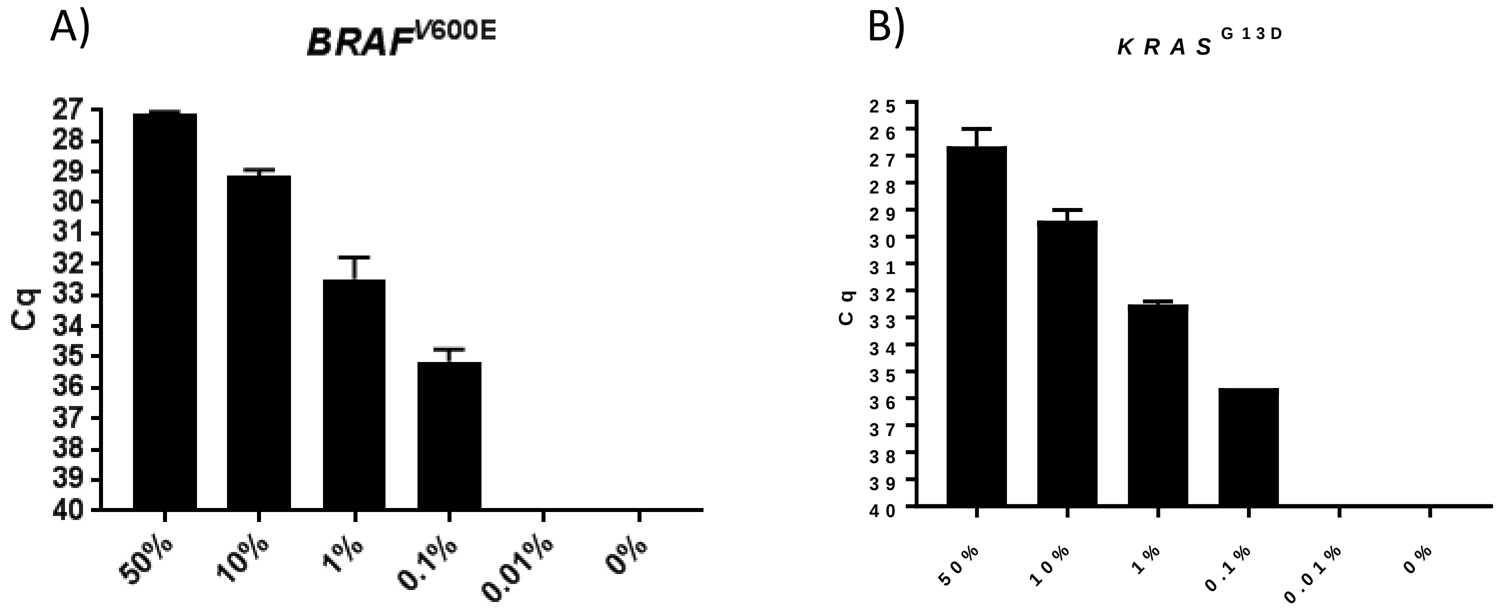

Suppl. Fig 1. : Analytical sensitivity of allele-specific PCR. A) Genomic DNA samples with decreasing A) *BRAF* V600E *or B) KRAS* G13D allelic frequency were used to determine the limit of detection of the allele-specific quantitative PCR (AS-QPCR). Amplification signal was expressed as Cq value.

Title

Isolation of extracellular vesicles improves the detection of mutant DNA from plasma of metastatic melanoma patients

Davide Zocco[1*], Simona Bernardi[4,5], Mauro Novelli[2,3], Chiara Astrua[2,3], Paolo Fava[2,3], Natasa Zarovni[1] Francesco M Carpi[1], Laura Bianciardi[1], Ottavia Malavenda[2,3], Pietro Quaglino[2,3], Chiara Foroni[4,5] Domenico Russo[4,5], Antonio Chiesi[1], Maria Teresa Fierro[2,3]

Supplementary Fig. 2

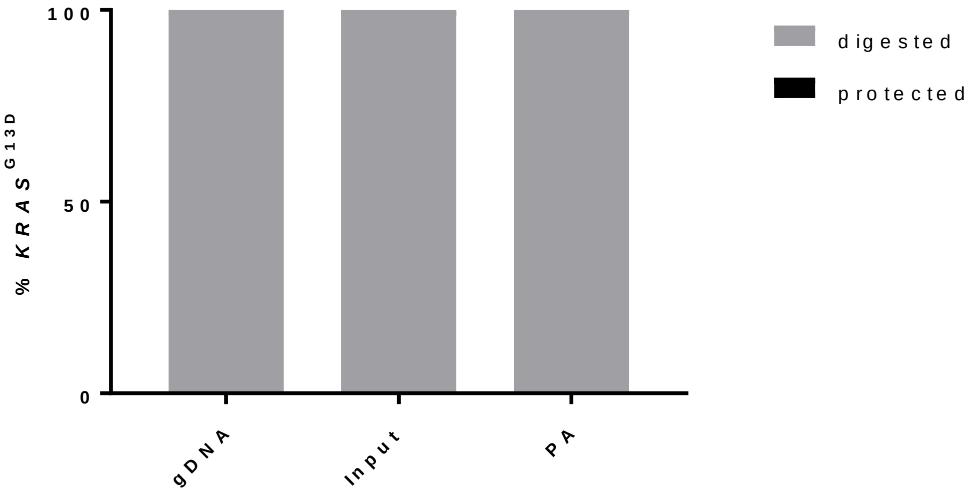

Suppl. Fig. 2: Dnase I protection assay on *KRAS* G13D –positive gDNA spiked after EV isolation. Following digestion, DNA was extracted and used to detect mutant *KRAS* by AS-QPCR. No mutation signal was observed after digestion, indicating the complete digestion of unprotected genomic DNA.

Title

Isolation of extracellular vesicles improves the detection of mutant DNA from plasma of metastatic melanoma patients

Davide Zocco[1*], Simona Bernardi[4,5], Mauro Novelli[2,3], Chiara Astrua[2,3], Paolo Fava[2,3], Natasa Zarovni[1] Francesco M Carpi[1], Laura Bianciardi[1], Ottavia Malavenda[2,3], Pietro Quaglino[2,3], Chiara Foroni[4,5] Domenico Russo[4,5], Antonio Chiesi[1], Maria Teresa Fierro[2,3]

Supplementary Fig. 3

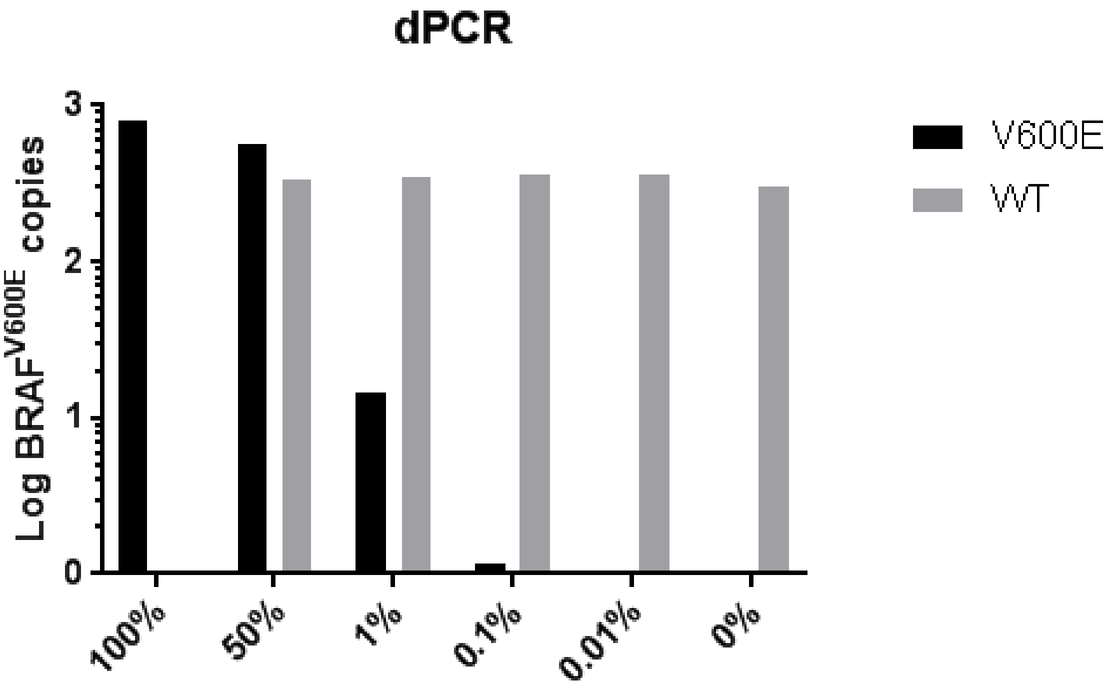

Suppl. Fig. 3: Analytical sensitivity of digital PCR for detection of *BRAF* gene copy number. A) Genomic DNA samples with decreasing *BRAF* ^{V600E} allelic frequency were used to determine the limit of detection of chip-based digital PCR. Results were expressed as gene copies on a logaritmic scale.

Title

Isolation of extracellular vesicles improves the detection of mutant DNA from plasma of metastatic melanoma patients

Davide Zocco[1*], Simona Bernardi[4,5], Mauro Novelli[2,3], Chiara Astrua[2,3], Paolo Fava[2,3], Natasa Zarovni[1] Francesco M Carpi[1], Laura Bianciardi[1], Ottavia Malavenda[2,3], Pietro Quaglino[2,3], Chiara Foroni[4,5] Domenico Russo[4,5], Antonio Chiesi[1], Maria Teresa Fierro[2,3]

Supplementary Fig. 4

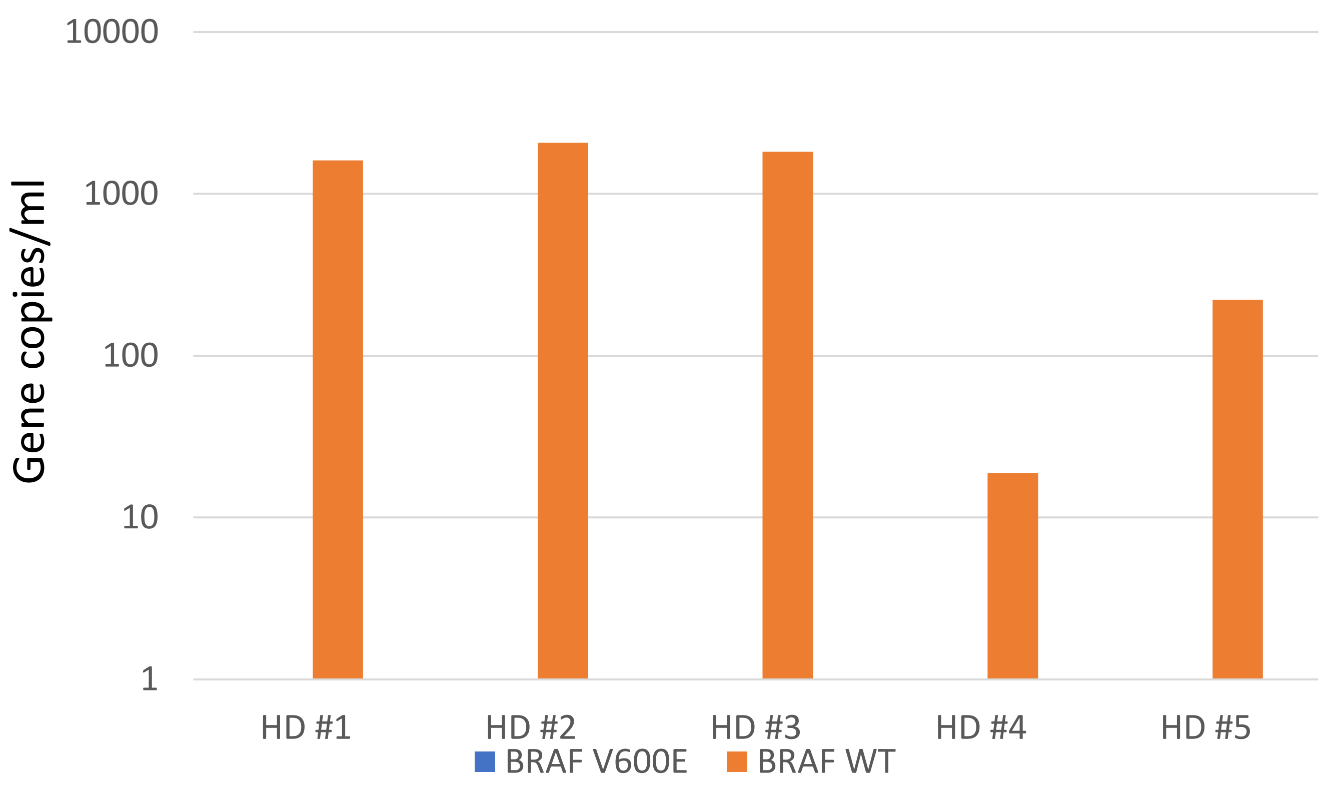

Supplementary Figure 4. Detection of *BRAF* mutant and wild type gene in the plasma of healthy donor patients. Plasma samples from healthy donor samples were processed after PA isolation and *BRAF*^{V600E} (blue) and *BRAF*^{WT} (orange) gene copies were detected by digital PCR. *BRAF* $^{V600E/WT}$ gene copies per ml of plasma was expressed on a logarithmic scale

Title

Isolation of extracellular vesicles improves the detection of mutant DNA from plasma of metastatic melanoma patients

Davide Zocco[1*], Simona Bernardi[4,5], Mauro Novelli[2,3], Chiara Astrua[2,3], Paolo Fava[2,3], Natasa Zarovni[1] Francesco M Carpi[1], Laura Bianciardi[1], Ottavia Malavenda[2,3], Pietro Quaglino[2,3], Chiara Foroni[4,5] Domenico Russo[4,5], Antonio Chiesi[1], Maria Teresa Fierro[2,3]

Supplementary Fig. 5

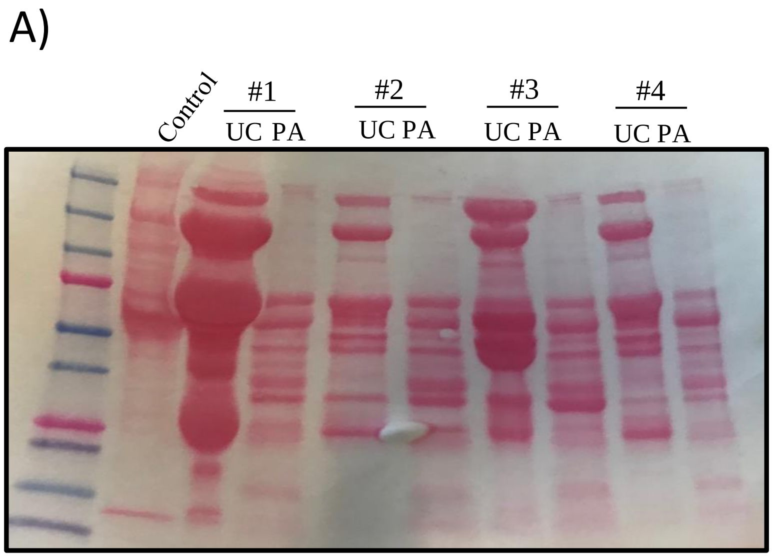

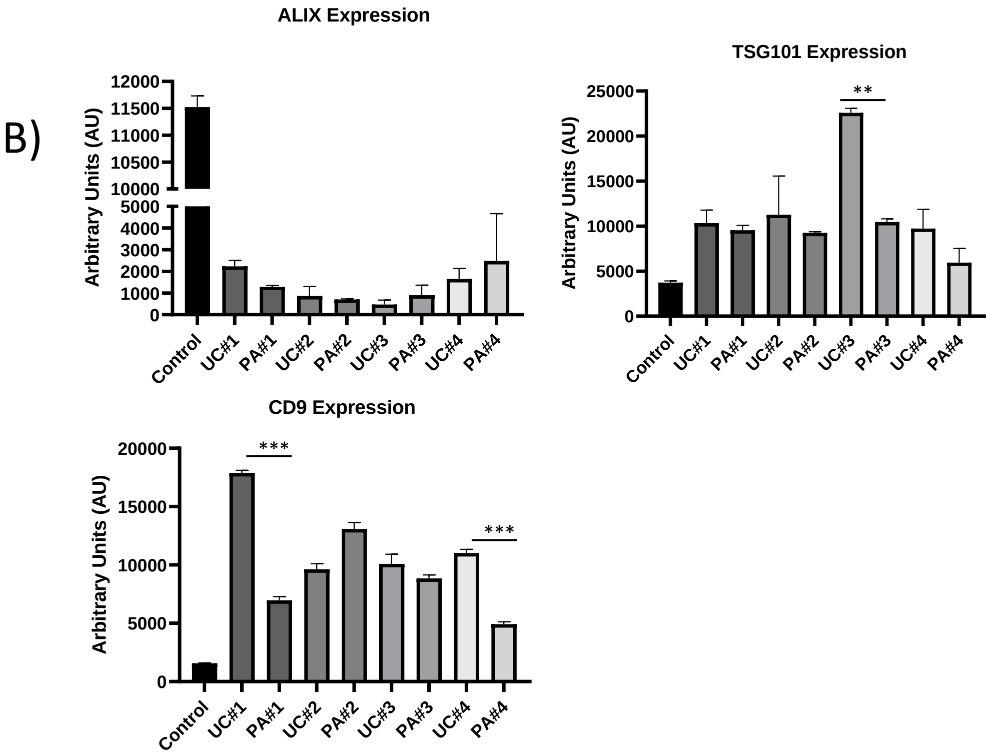

Suppl. Figure 5: Ultracentrifugation (UC) yields samples with higher protein load but comparable EV content than peptide-affinity (PA) isolation from plasma samples of metastatic melanoma (MM) cancer patients.
A) One ml of plasma samples from MM patients were either processed by UC or PA. Equal sample volumes were loaded onto an SDS page system and stained with Red Ponceau for general protein staining. B) Densitometric analysis of Alix, TSG101 and CD9 expression from western blot bands using ImageJ software. Statistical analysis was performed between UC and PA samples using paired parametric t-test (** $p<0.005$; *** $p<0.0005$).

Title

Isolation of extracellular vesicles improves the detection of mutant DNA from plasma of metastatic melanoma patients

Davide Zocco[1*], Simona Bernardi[4,5], Mauro Novelli[2,3], Chiara Astrua[2,3], Paolo Fava[2,3], Natasa Zarovni[1] Francesco M Carpi[1], Laura Bianciardi[1], Ottavia Malavenda[2,3], Pietro Quaglino[2,3], Chiara Foroni[4,5] Domenico Russo[4,5], Antonio Chiesi[1], Maria Teresa Fierro[2,3]

Supplementary Fig. 6

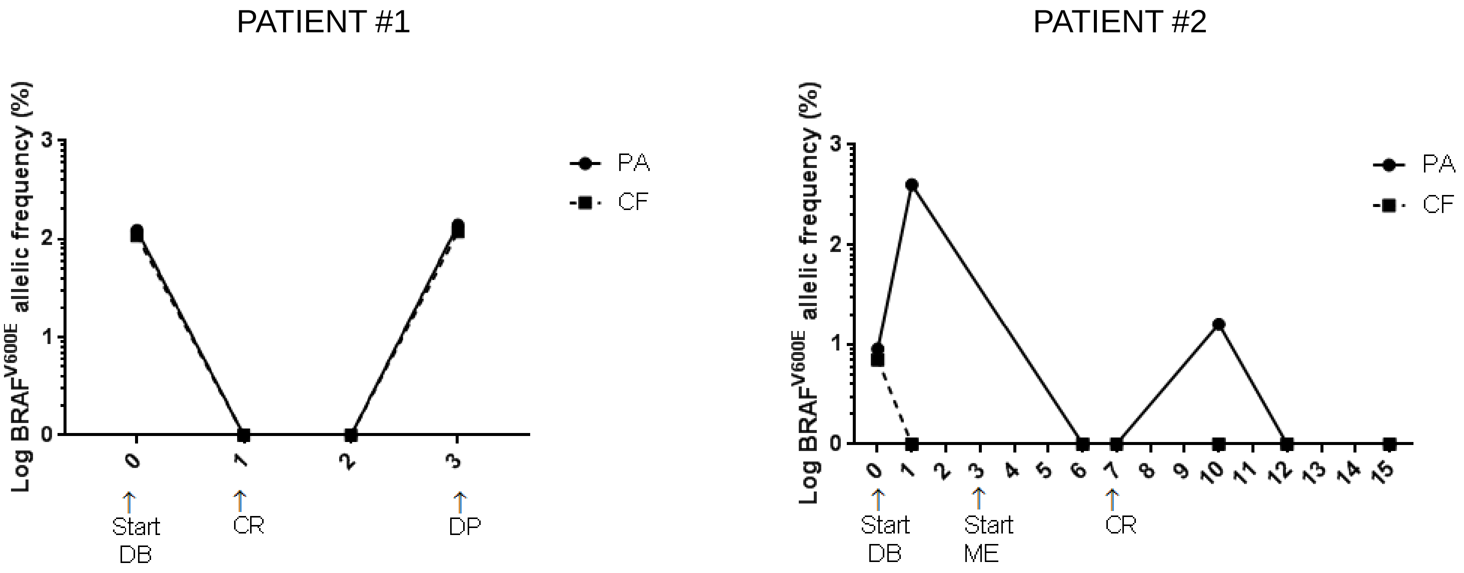

Supplementary Figure 6. Monitoring *BRAF* ^{V600E} allelic frequency during BRAF inhibitor (BRAFi) treatment. Plasma samples from high- and low-mutation burden were collected at the beginning and during BRAFi treatment. Samples were processed after PA or CF-isolation and *BRAF*$^{V600E/WT}$ gene copies were detected by digital PCR. *BRAF* ^{V600E} allelic frequency was expressed on a logarithmic scale. DB = Dabrafenib; ME = Mekinist; CR = complete response; DP = disease progression.

Title

Isolation of extracellular vesicles improves the detection of mutant DNA from plasma of metastatic melanoma patients

Davide Zocco[1*], Simona Bernardi[4,5], Mauro Novelli[2,3], Chiara Astrua[2,3], Paolo Fava[2,3], Natasa Zarovni[1] Francesco M Carpi[1], Laura Bianciardi[1], Ottavia Malavenda[2,3], Pietro Quaglino[2,3], Chiara Foroni[4,5] Domenico Russo[4,5], Antonio Chiesi[1], Maria Teresa Fierro[2,3]

Supplementary Fig. 7

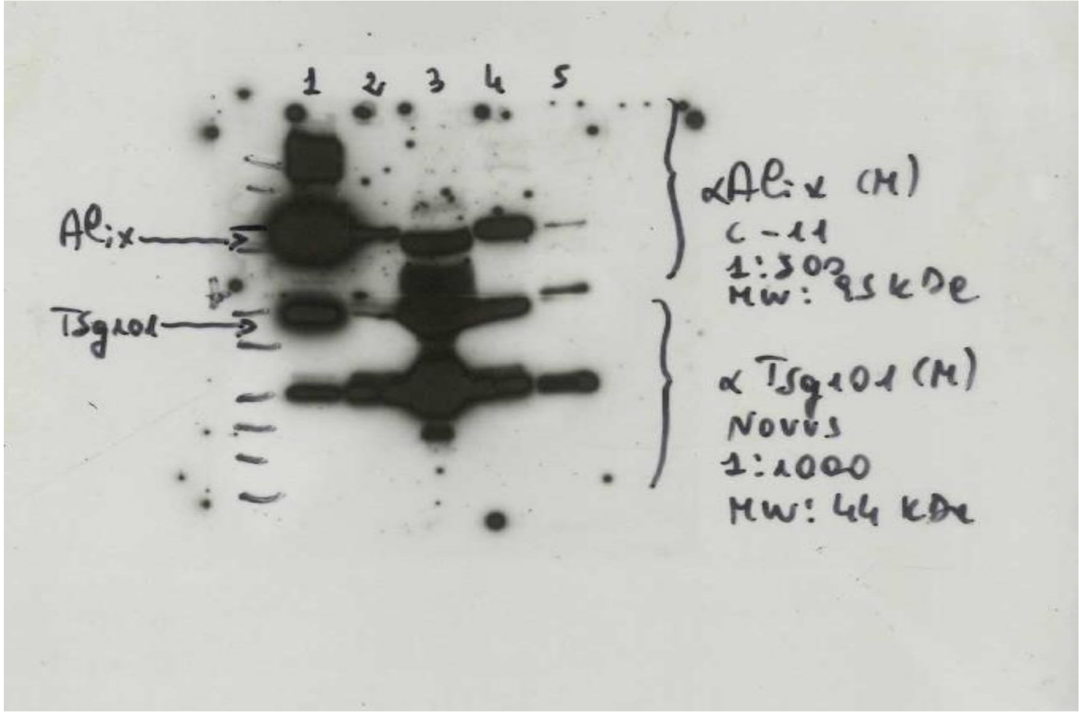

Supplementary Figure 7. Original blot of cropped western blot analysis shown in Figure 1C
